# Novel Internet Advertising Approach to Raise Public Awareness About Metabolic Dysfunction‐Associated Steatotic Liver Disease

**DOI:** 10.1002/jgh3.70436

**Published:** 2026-06-20

**Authors:** Kaori Inoue, Kaoru Araki, Kaoru Shibayama, Tomomi Yada, Nagisa Hara, Satoshi Oeda, Kazuma Udo, Shohei Tobu, Shun Yamashita, Hiroshi Isoda, Yuichiro Eguchi, Keizo Anzai, Mitsuru Noguchi, Hirokazu Takahashi

**Affiliations:** ^1^ Liver Center, Saga University Hospital Saga Japan; ^2^ Education and Research Center for Community Medicine, Faculty of Medicine Saga University Saga Japan; ^3^ Office for Equality, Diversity, and Inclusion, Saga University Saga Japan; ^4^ Health Care Center, Saga University Saga Japan; ^5^ Institute of Nursing, Faculty of Medicine Saga University Saga Japan; ^6^ Loco Medical General Institute Saga Japan; ^7^ Department of Laboratory Medicine Saga University Hospital Saga Japan; ^8^ Department of Urology, Faculty of Medicine Saga University Saga Japan; ^9^ Division of Diabetes, Endocrinology, Hepatology Center Takagi Hospital Fukuoka Japan; ^10^ Division of Metabolism and Endocrinology, Faculty of Medicine Saga University Saga Japan

**Keywords:** banner advertisement, click‐through rate, co‐occurrence term, metabolic dysfunction‐associated steatotic liver disease, search history

## Abstract

**Background and Aims:**

Metabolic dysfunction‐associated steatotic liver disease (MASLD), causing cirrhosis and liver cancer, is prevalent worldwide. Analyses of internet searches for “fatty liver” and methods to raise MASLD awareness are needed.

**Methods:**

We retrospectively analyzed co‐occurring terms with “fatty liver” and tracked search histories to identify key terms. In a comparative study, a banner advertisement about MASLD was shown to individuals searching for key terms (key term group: KTG) or regardless of the search terms (control group: CTR). The ad redirected viewers to an educational cartoon, followed by a questionnaire.

**Results:**

The most frequent co‐occurrence term was “how to treat.” People searched mainly for lifestyle‐related disease and diet terms. Key terms included “diet,” “blood sugar level,” “cholesterol,” “triglyceride,” “visceral fat,” and “antihypertensive drugs.” Banner views were 5 864 184 for KTG and 49 388 176 for CTR. KTG had a higher click‐through rate than CTR (0.097% vs. 0.063%; *p* < 0.001) and more survey responses (3.22% vs. 0.84%; *p* < 0.001). KTG participants also had a greater intention to seek medical care early (60.2% vs. 42.4%; *p* = 0.003).

**Conclusion:**

Search term‐based advertising may help increase public awareness of MASLD and may encourage earlier intention to seek medical consultation.

**Abbreviations**
ALD: alcohol‐related fatty liver disease
CTR: control group
KTG: key term group
MASLD: metabolic dysfunction‐associated steatotic liver disease
MetALD: metabolic dysfunction and alcohol‐associated liver disease

## 1 Introduction

Proliferation of the internet has changed the life of humans worldwide. Infodemiology is an academic field that involves the use of information derived from online sources, particularly from the internet, to understand and analyze the patterns related to health and disease in the general public [1]. Using an infodemiological approach, the interest and behavior of people regarding diseases, treatments, and health care systems can be surveyed. Regarding infectious diseases such as COVID‐19, the prevalence and analysis of seasonal patterns can be predicted by examining the correlation between internet search data and actual incidence rates. Accordingly, predictive models for outbreaks, seasonal patterns, and prescriptions have been developed [2].

Metabolic dysfunction‐associated steatotic liver disease (MASLD) [3], formerly known as nonalcoholic fatty liver disease, is a chronic liver disease with an approximately 30% prevalence globally [4]. MASLD is concomitant with cardiometabolic risk, including overweight, diabetes, dyslipidemia, and hypertension. Therefore, patients with MASLD have various diseases, including cardiovascular disease and extra‐hepatic cancers related to obesity and metabolic dysfunction, as well as liver cirrhosis and liver cancer [5, 6, 7].

Analyses of internet searches performed by the general public targeting obesity and lifestyle diseases have been reported. World Diabetes Day is an annual international day of observance dedicated to raising awareness about diabetes. The volume of internet searches related to diabetes increases up to World Diabetes Day and rapidly decreases during the following week [8]. Internet searches regarding diet increase during the holiday season, and raising awareness against obesity is considered to be effective around this season [9, 10]. Infodemiological analysis is beneficial for lifestyle‐related diseases as well as infectious diseases. Moreover, in terms of social implementation of an infodemiological approach targeting the general public, using an active approach such as an internet advertising strategy to raise awareness about a particular disease could be effective. Regarding MASLD, related internet searches and the effect of an internet advertising approach have not been well studied. We hypothesized that an intervention using an educational advertisement targeting individuals with a potential interest in MASLD will contribute to enhancing their intention to receive medical care if they conduct an internet search. We aimed to retrospectively analyze the history of internet searches for “fatty liver” and identify the key search terms as an interventional point. We also conducted a comparative study to evaluate whether an educational advertisement would increase the intention to seek medical care for fatty liver among the general public.

## 2 Materials and Methods

### 2.1 Analysis of Internet Searches

To analyze internet searches, we used the web application software DS.INSIGHT (https://ds.yahoo.co.jp/) (LY Corporation, Tokyo). Regarding particular search terms, analysis of the search volume, analysis according to age, sex, and region, analysis of co‐occurrence terms, and analysis of the search history before and after a search for a particular term can be performed. Analysis using DS.INSIGHT is based on internet search data from Yahoo! JAPAN (https://www.yahoo.co.jp/). Because Yahoo! JAPAN is a Japanese portal site, search terms are mostly in Japanese, and the data acquired from DS.INSIGHT are in Japanese. All data and results were translated from Japanese into English.

### 2.2 Analysis of Co‐Occurrence Terms With “Fatty Liver”

In general, people use multiple terms in internet searches. Co‐occurrence terms used in searches for “fatty liver” from January 1, 2020 to December 31, 2020 were retrospectively investigated using the co‐occurrence term detection function in DS.INSIGHT. The most commonly searched co‐occurrence terms and “fatty liver” were used for analysis of search term history as follows. Network analysis of the co‐occurrence terms was performed using DS.INSIGHT.

### 2.3 Analysis of Search Term History and Definition of Key Terms

From 5 months prior to searches conducted using the keyword “fatty liver” and the most commonly searched co‐occurring term, we retrospectively analyzed the history of search terms using the time‐series function in DS.INSIGHT from January 1, 2020 to December 31, 2020. All extracted search terms within the study period were reviewed and grouped based on medical knowledge and similarities in their content by two investigators (K.I. and H.T.). The thematic categories were determined through discussion and consensus between the investigators. We then analyzed temporal changes in the proportion of each category with time‐series analysis. Among these categories, the category that accounted for the largest proportion of both search volume and number of terms, and that also showed the highest proportion in the time‐series analysis, was selected for further analysis. Six key terms were selected based on the search volume and their presumed medical relevance to fatty liver.

### 2.4 Comparative Study Using Banner Advertisement and Questionnaire Survey

The study design of the comparative study and questionnaire survey are summarized in Figure S1. From January 10 to January 18, 2022, our internet banner advertisement appeared in search results when users aged 40–59 years searched for any of the predefined key terms (key term group; KTG). For the control group (CTR), the same advertisement was distributed to users aged 40–59 years, both male and female, who were categorized under the audience category “Diet and Health” in the Yahoo! JAPAN advertising system, irrespective of the search terms used. In this study, to avoid a markedly low click‐through rate, the target population was restricted to users aged 40–59 years, both male and female, and the CTR was further defined as those categorized under the “Diet and Health” audience segment. This approach was based on the assumption that individuals aged 40–59 years have relatively high levels of internet activity and that users in the “Diet and Health” audience segment are more likely to show interest in the banner advertisements used in this study. This audience category was defined according to Yahoo! JAPAN's user segmentation system.

After clicking the banner advertisement, users in both groups were directed to an educational cartoon about MASLD. The cartoon consisted of eight panels and featured a 60‐year‐old obese female character. It presented a narrative explaining the association between MASLD and serious liver diseases, including liver cirrhosis and liver cancer. Through the storyline, the cartoon emphasized the importance of medical consultation and lifestyle modification for the prevention and management of MASLD. The cartoon was created by a professional illustrator and medically supervised by hepatology specialists (K.I, S.O, H.I, and HT) to ensure the accuracy of the information. Following the cartoon, a questionnaire survey was provided that asked two questions about the viewer's intention to visit a medical provider. The click‐through rate (click/impression) for the banner advertisement, the response rate for the questionnaire survey, and responses to the questionnaire were compared between the two groups.

At the stage when the banner advertisement was displayed, individuals were not yet enrolled in the study; however, the banner clearly indicated that it was issued by the Liver Center, Saga University Hospital. After clicking the banner, users were informed that they would be shown an educational cartoon and were asked whether they wished to participate in a study analyzing viewing behavior. They were also explicitly informed that a questionnaire survey would be conducted after viewing the cartoon and that their responses would be used for study inclusion. At the beginning of the questionnaire, participants were informed of the purpose of the questionnaire survey and instructed to proceed only if they consented to participate. Electronic informed consent was therefore obtained through voluntary completion and submission of the questionnaire. The survey and questionnaire were conducted anonymously, and participation was voluntary. No minors were included in this study. The study protocol was approved by the Institutional Review Board of Saga University Faculty of Medicine.

### 2.5 Statistical Analysis

Qualitative data are presented as number (percentage). Fisher's exact test was used to compare categorical variables. All statistical analyses were performed using JMP Pro 14 (SAS Institute Inc., Cary, NC, US). Differences were considered significant with *p* < 0.05.

## 3 Results

### 3.1 Co‐Occurrence Term Analysis

**FIGURE 1:**
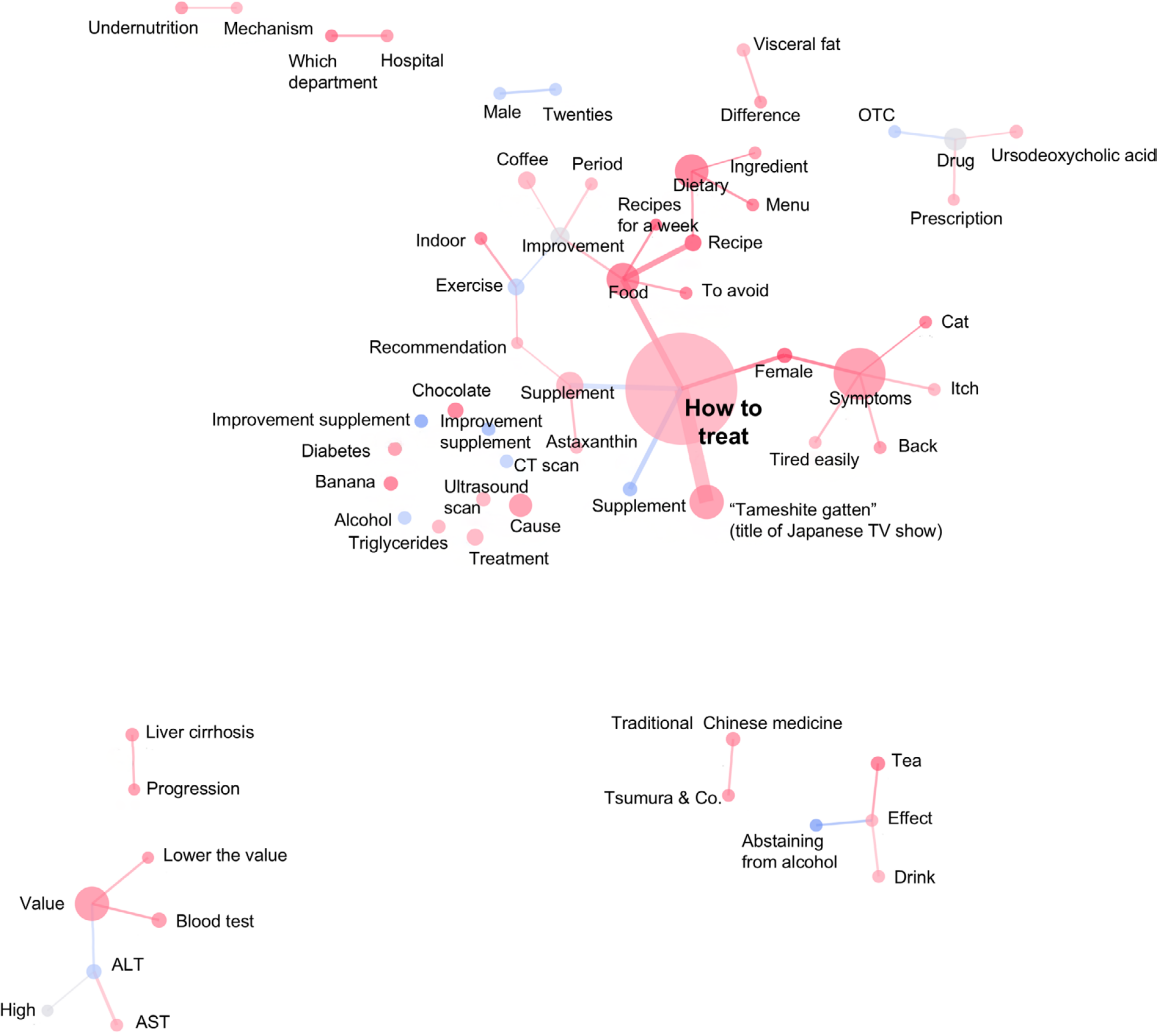
Network chart of co‐occurrence terms with “fatty liver.” Pink circle and blue circle represent female‐dominant and male‐dominant co‐occurrence terms. The size of the circle represents the search volume. The connection among individual terms represents the tendency to be searched for simultaneously. Images were obtained from DS. INSIGHT. ALT, alanine transaminase; AST, aspartate transaminase; CT, computed tomography; OTC, over the counter.

A network chart of co‐occurrence terms searched together with “fatty liver” is shown in Figure 1. Among the co‐occurrence terms, “how to treat” was most frequently searched. Searching for “how to treat” together with “fatty liver” was more frequent among female than male internet users. In the “how to treat”‐centered network, diet, supplementation, and television programs on healthy living were also searched. Independent of this network, “result value,” “visceral fat,” “diabetes,” “triglyceride,” and “drug” were the co‐occurrence terms and revealed the individual network; however, the volume and network of these were smaller than those of “how to treat.” Accordingly, we performed the following analysis with “fatty liver” and “how to treat.”

### 3.2 History of Search Terms Before Searching for “Fatty Liver” and “How to Treat”

**FIGURE 2:**
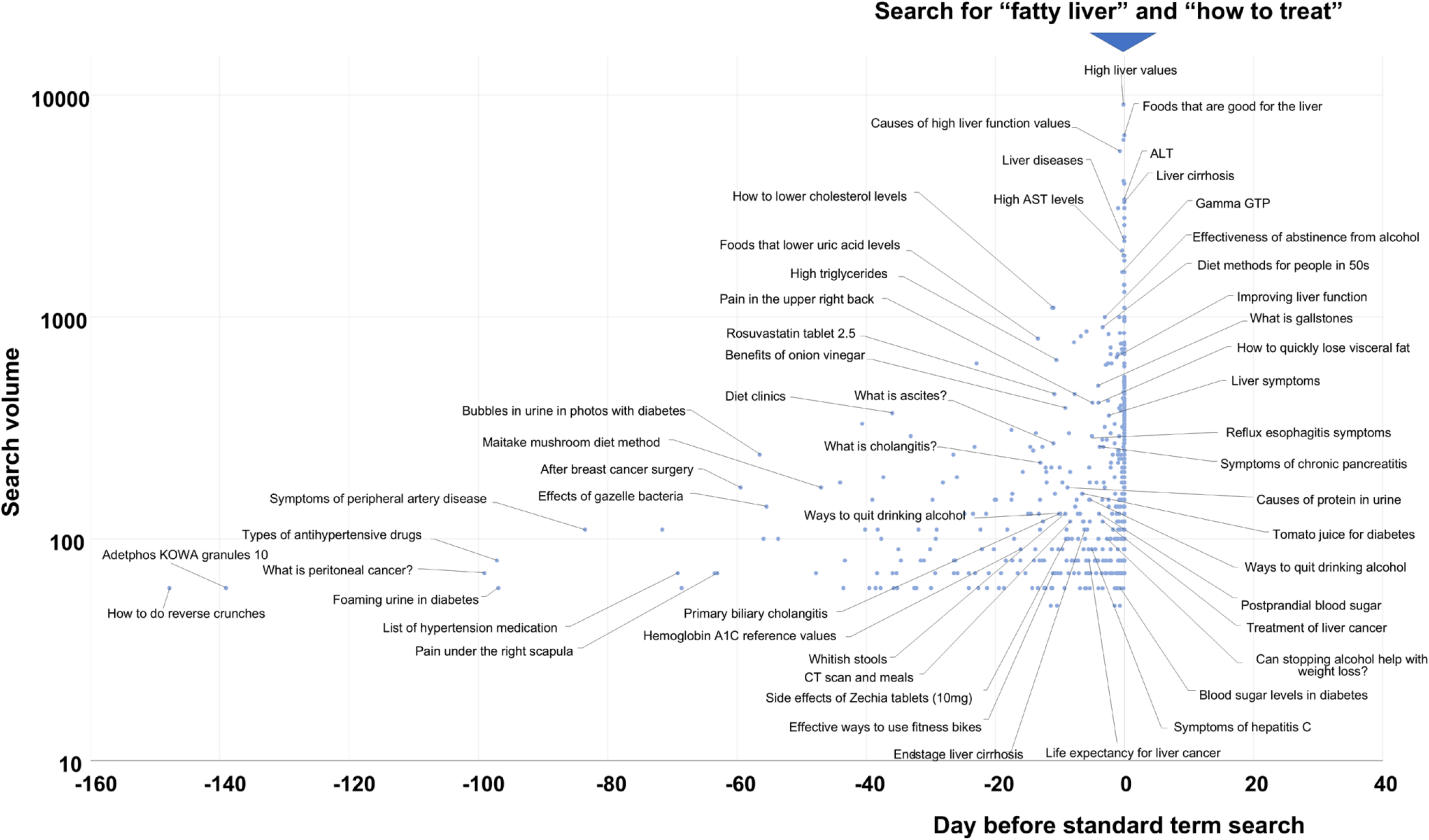
History of search terms prior to searching for “fatty liver” and “how to treat.” The 500 most frequently searched terms are shown in the graph. ALP, alkaline phosphatase; ALT, alanine transaminase; AST, aspartate transaminase; CT, computed tomography; GTP, glutamyl transpeptidase; LDL, low‐density lipoprotein.

Figure 2 shows a history of the search terms prior to searches using “fatty liver” and “how to treat.” History of search terms means that people who searched for these terms would search for “fatty liver” and “how to treat” in the future (Day 0). Terms associated with lifestyle‐related diseases, diet, and obesity such as diet method, hyper‐triglyceride, visceral fat, symptoms, supplements, over the counter drugs, traditional Chinese medicine, medical examinations, and liver diseases were also searched. The timing of searches and the search volume for all search terms are available in Table S2. Terms were classified into eleven categories: Lifestyle‐related diseases and diet, symptoms in the liver and upper abdomen, symptoms other than in the upper abdomen, extrahepatic diseases, laboratory tests and imaging examinations, liver diseases, pharmaceuticals, exercise and fitness, alcohol, supplements, and unclassified. Representable search terms for individual categories are shown in Table 1.

**TABLE 1:** Categorization of the search terms used prior to searching “fatty liver” and “how to treat.”

Category	Search terms					
Lifestyle‐related diseases and diet	How to lower **cholesterol** levels	**Diet** methods for people in their 50s	Postprandial **blood sugar**	How to quickly lose **visceral fat**	High **triglyceride**	Immediate treatment to lower **blood pressure**
Liver and upper abdomen symptoms	Reflux esophagitis symptoms	Liver symptoms	Pain from the right flank to the back	Discomfort on the right flank	What is ascites?	Pain on the left side of the epigastric region
Symptoms other than in the upper abdomen	Red palms	Pain in the upper right back	Dark urine color	Causes of protein in urine	Diseases that cause foamy urine	Whitish stool
Extrahepatic diseases	What are gallstones?	Symptoms of kidney cancer	Symptoms of bile duct cancer	Symptoms of chronic pancreatitis	Symptoms of kidney stones	What is cholangitis?
Laboratory tests and imaging examinations	Liver function values	What is ALP?	What can be diagnosed with abdominal echography?	Health checkups and re‐examinations	CT scan and meals	Hemoglobin A1C reference values
Liver diseases	Improving liver function	Primary biliary cholangitis	Treatment of liver cancer	End‐stage liver cirrhosis	Symptoms of hepatitis C	Life expectancy for liver cancer
Pharmaceuticals	*Rosuvastatin* tablet 2.5 mg	Side effects of *Bofutsusho‐san* (herbal medicine)	*Tsumura* 25 effects (herbal medicine)	Side effects of *Zechia* tablets (10 mg)	*Tsumura* 107 *Goshajinkigan* (herbal medicine)	Effectiveness of *Tsumura* 62 (herbal medicine)
Exercise and fitness	Effective ways to use fitness bikes	How to do reverse crunches	How long does it take to see results from walking?	—	—	—
Alcohol	Effectiveness of abstinence from alcohol	Benefits of abstaining from alcohol	Ways to quit drinking alcohol	Abstinence from alcohol	Withdrawal symptoms from alcohol	Can stopping alcohol help with weight loss?
Supplements and unbalanced meal	*Naisi support*	Reviews of *Kaitousha* tea	Benefits of onion vinegar	Benefits of clams	Tomato juice for diabetes	*Urashirogashi* tea
Unclassified	Insurance coverage for patients with cancer	Dress code for niece's wedding	Good health using feng shui	—	—	—

*Note:* Words in italics are proper names of product or company. Words in bold are defined as key terms in the prospective survey.

Abbreviations: ALP, alkaline phosphatase; CT, computed tomography.

### 3.3 Changes in the Proportion of Search Term Categories

**FIGURE 3:**
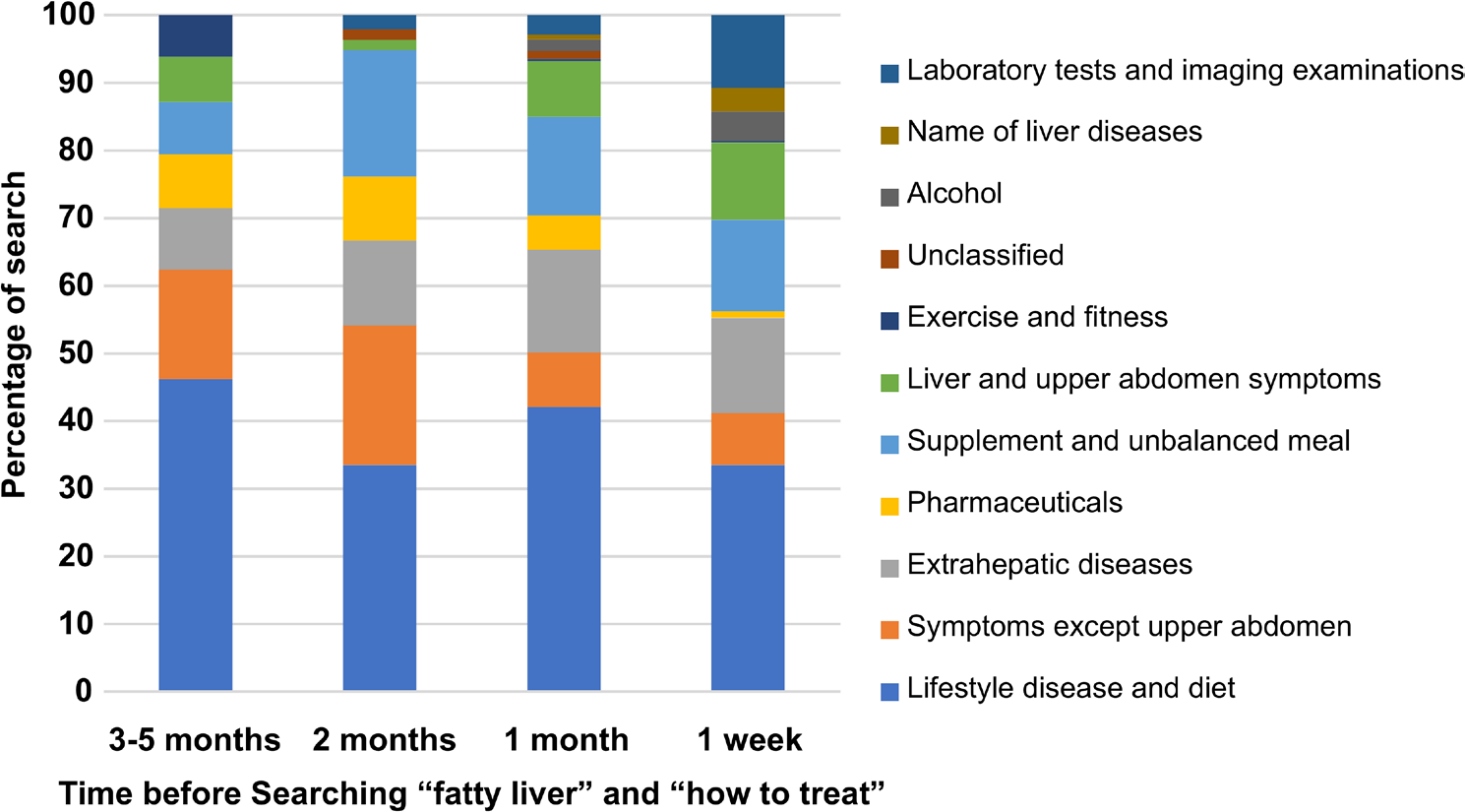
Changes in the proportion of search term categories. Proportion of individual categories changed prior to searching for “fatty liver” and “how to treat.”

The proportions for each search term category are shown in Figure 3. From 3–5 months to 1 week before searches using “fatty liver” and “how to treat,” terms in the category of lifestyle‐related diseases and diet were the most frequently searched, although the proportion decreased as Day 0 neared (46.2% at 5–3 months prior to Day 0, 33.5% at 2 months prior, 42.0% at 1 month prior, and 33.5% at 1 week prior to Day 0). However, the proportions at these timepoints for terms in the category of extrahepatic diseases (9.1%, 12.1%, 15.1%, and 17.1%), as well as terms related to laboratory tests and imaging examinations (0.0%, 3.0%, 5.9%, and 12.6%), increased continuously over the study period.

### 3.4 Prospective Survey With Banner Advertisement

**FIGURE 4:**
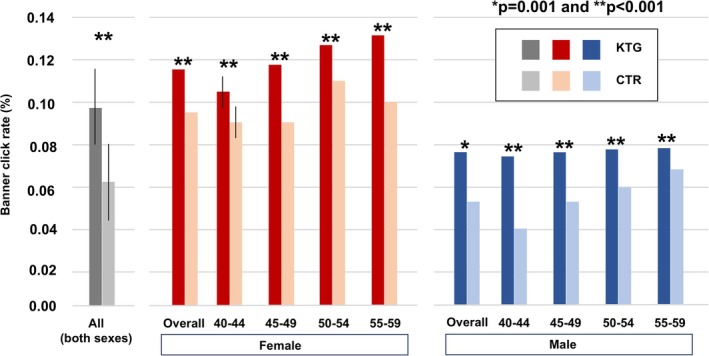
Click‐through rate for banner advertisement. Click‐through rate for the banner advertisement compared between the KTG and CTR groups. *p = 0.001 and **p < 0.001 using Fisher's exact test. CTR, control; KTG, key term group.

Among the categories, “lifestyle‐related diseases and diet” which accounted for the largest proportion of both search volume and number of terms and also showed the highest proportion in the time‐series analysis, was selected for further analysis. Based on the search volume during the observation period, top six words were selected as key terms: “cholesterol” (search volume = 115 800), “diet” (101300), “blood sugar level” (73900), “visceral fat” (66900), “triglyceride” (63300) and “antihypertensive drugs” (37900) (Table 1). These words were considered to be strongly relevant to fatty liver. The banner advertisement was shown to people who searched for the key terms, defined as the KTG group, (viewed 5 864 184 times) and to the CTR group (viewed 49 388 176 times), shown in Table S1. In the overall population, the KTG group had a significantly higher click‐through rate for the banner advertisement than the CTR group (KTG, 0.097% and CTR, 0.063%; *p* < 0.001) (Figure 4). The difference was also significant between female internet users (KTG, 0.118% and CTR, 0.095%; *p* < 0.001) and male users (KTG, 0.076% and CTR 0.053%; p < 0.001). When the click‐through rate was compared between groups stratified by age (Age 40–44 years, 45–49 years, 50–54 years, and 55–59 years), the KTG group showed a significantly higher click‐through rate than the CTR group.

### 3.5 Questionnaire Survey

**FIGURE 5:**
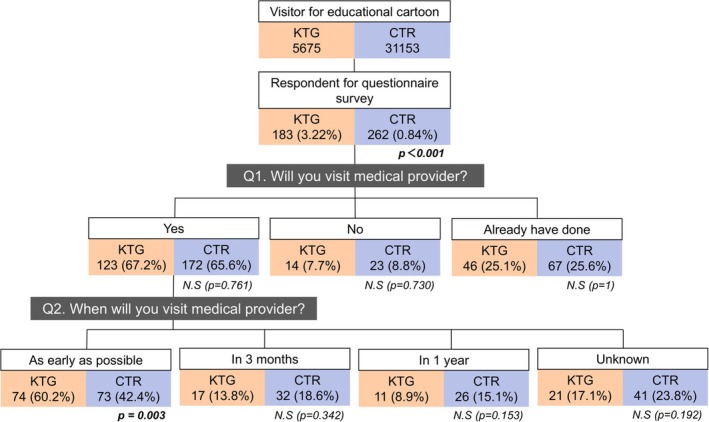
Questionnaire survey following the educational cartoon. *p* value using Fisher's exact test. CTR, control; KTG, key term group.

The questionnaire survey was viewed by 5675 people in the KTG group and 31 153 people in the CTR group (Figure 5). Responses to the questionnaire were obtained from 183 people (3.22%) in the KTG group and 262 people (0.84%) in the CTR group, with a significant difference in the response rate (*p* < 0.001). Regarding the first question, which queried the intention to visit a medical provider, 123 people (67.2%) in the KTG group and 172 people in the CTR group (65.6%) stated that they would visit a medical provider, with no significant difference between the groups (*p* = 0.761). For those who answered “yes” to the first question, a second question was asked, related to when they intended to visit a medical provider. The response “as early as possible” was more frequent in the KTG group than in the CTR group (74 people, 60.2% vs. 73 people, 42.4%, respectively; *p* = 0.003).

## 4 Discussion

In the present study, we analyzed internet searches conducted by the Japanese general public for the term “fatty liver.” Among the terms frequently searched prior to searching for “fatty liver” and “how to treat,” six key terms including “diet,” “blood sugar level,” “cholesterol,” “triglyceride,” “visceral fat,” and “antihypertensive drugs” were chosen for this prospective study. A banner advertisement regarding MASLD, which appeared after a search for any of the six key terms, successfully showed a significantly higher click‐through rate than the rate for a banner advertisement delivered irrespective of the search queries. Via our investigation of the search history prior to searches for “fatty liver,” we could potentially target people who might be interested in learning more about “fatty liver.” Moreover, the questionnaire survey demonstrated that targeted individuals were willing to cooperate with the questionnaire survey and were motivated to receive medical care as early as possible. To our knowledge, this is the first comparative study using targeting advertisement based on the analysis of internet search history. Our findings can contribute to identifying people who are potentially concerned about MASLD and might accelerate the receipt of medical care by those who need it.

In accordance with the increasing prevalence of MASLD [11], the internet search volume regarding this topic is increasing globally [12, 13]. However, the level of interest regarding fatty liver among the general public has not been elucidated. In patients with MASLD, data obtained from social network services (SNS), including X (formerly known as Twitter), have recently been analyzed [14, 15]. Lazarus et al. reported that the most frequent mention by patients with MASLD was related to ongoing management of MASLD, including the topics of lifestyle, treatment, and health care providers [14]. In that study, diagnosis/tests, signs/symptoms, and cause/risk factors of MASLD were also frequently mentioned. Compared with internet searches performed in our study, SNS activity regarding MASLD could indicate more active motivation among users, and interest could differ between the general public and patients with MASLD. In the present study, the most frequently searched term with “fatty liver” was “how to treat,” suggesting that the general public has concerns about the management and treatment of “fatty liver,” similar to patients with MASLD. However, in the history of terms searched prior to searches for “fatty liver,” terms associated with lifestyle‐related diseases, including the risk factors of MASLD, were most frequent in this study. For the diagnosis of MASLD, patients should meet at least one cardiometabolic criterion: Overweight, glucose intolerance, hypertension, and hypertriglycemia or low high‐density lipoprotein cholesterolemia [3]. In the present study, people who would be interested in “fatty liver” are probably highly concerned about lifestyle‐related diseases and lifestyle factors, including obesity, diabetes, dyslipidemia, and hypertension. Therefore, by raising public awareness, people who are concerned about these cardiometabolic criteria might understand that they may have MASLD.

In terms of education for patients and the general public, the quality of information on the internet is important. Moreover, the information required varies depending on the person conducting the internet search and their medical condition. The language spoken and understood by the person who is conducting an online search and the language of websites also affect the usefulness of the provided information. A number of reports have indicated that websites focusing on particular diseases are of poor quality and are not informative for patients. So et al. performed a website survey in Korea and reported that online information for patients with MASLD was generally poor quality and missing important topics, regardless of the site administrator [16]. Regarding other diseases and conditions such as obesity, diabetes, inflammatory bowel disease, and cancer, websites are considered to be helpful from the viewpoint of patients and families who require detailed information, including regarding therapy and medical providers [17, 18, 19, 20]. However, information for the general public about particular diseases may be generalized and basic. In our study, people who searched for any of the six key terms were potentially interested in MASLD or at risk for MASLD. Therefore, intervention for this target population should provide guidance to find appropriate websites that provide basic and essential information, to encourage awareness about MASLD and accelerate behavioral changes, leading to individuals having greater involvement in their medical care. Our results indicated that web banner advertisements are a useful tool to promote these steps.

In the present study, key terms were determined to check the medical relevance to MASLD and a cartoon was used in the banner advertisement, followed by educational information that was friendly and easy to understand by the general public. Online advertisements can be personalized with viewer targeting. However, a number of items must be determined during the process of creating such advertisements, including the platform to be used (e.g., conventional website, SNS, and YouTube), equipment used by the viewer (e.g., smart phone, tablet, and personal computer), color of the content (monochrome or color), style of the advertisement (e.g., text, still image, and movie), and the tagline [21, 22]. Recently, neural network analysis and artificial intelligence have been used to predict and increase click‐through rates [23, 24]. To effectively enhance awareness about a particular disease, optimization of advertisements using these technologies is important.

Internet‐based information seeking has been increasing globally and in Japan [25], and it is considered a highly useful medium for public health awareness. Compared with so‐called display advertising such as banner ads, advertisements linked to search behavior have been reported to yield higher CTR [26] [X1], and our findings were consistent with this. In the present study, assuming one million impressions, the CTR was 0.097% in the key term group (KTG) versus 0.063% in the CTR (*p* < 0.001), corresponding to approximately 970 clicks in the KTG group and 630 clicks in the CTR—about a 1.5‐fold increase. Although this approach targets a broad population and the overall response rate is not high, it allows relatively simple outreach at scale and enables engagement with individuals who may have latent interest in the disease. Therefore, this strategy may represent a useful method for public health awareness. On the other hand, in this study, exposure to the banner advertisement and the questionnaire results indicated a positive intention to seek medical care; however, we did not evaluate actual healthcare‐seeking behavior, such as visits to medical institutions. Because changes in behavioral intention do not necessarily translate into actual behavior, further studies incorporating more objective and behavior‐proximal indicators are needed to assess the impact of our intervention on real‐world health behavior.

The present study had several limitations. First, as shown in Figure 3, alcohol‐related terms accounted for a certain proportion of searches associated with “fatty liver” and “how to treat.” In addition, it is plausible that some individuals with drinking habits may not perform such searches. Therefore, our findings cannot be generalized to steatotic liver diseases related to alcohol use, including alcohol‐associated liver disease (ALD) and metabolic dysfunction and alcohol‐associated liver disease (MetALD). When targeting alcohol‐related steatotic liver disease, optimization of key term selection and banner advertisement strategies would be necessary. Moreover, the desired behavioral outcomes would extend beyond access to healthcare or social resources to include abstinence or reduction in alcohol consumption. Further studies specifically focusing on ALD and MetALD are warranted. Second, from the perspectives of scientific reproducibility and generalizability, this study was conducted in Japan using Japanese‐language search terms, and the target population was restricted to individuals aged 40–59 years; in addition, the educational material featured a 60‐year‐old woman. Given that access to and use of web‐based information vary by age and sex [25], the applicability of these findings to other languages, age groups, and populations remains uncertain. Third, the categorization was based on search volume and supplemented by medical judgment, rather than relying solely on an automated clustering method; therefore, it may limit reproducibility. Furthermore, the CTR was defined using the “Diet and Health” audience segment in the Yahoo! JAPAN advertising system, for which the categorization method is proprietary and was not disclosed. This may have introduced selection bias and also limits the reproducibility of the study. Fourth, our prospective survey using a banner advertisement and questionnaire was conducted after the start of the COVID‐19 pandemic, changes in internet use owing to the pandemic might affect the results [27].

In conclusion, we analyzed the co‐occurrence terms for fatty liver and the search history before searches for the term “fatty liver” in Japan Search term–based advertising may help increase public awareness of MASLD and may encourage earlier intention to seek medical consultation.

## Funding

This research was supported by MEXT support program for the development of human resources in science and technology initiative for realizing diversity in the research environment and Health, Labour, and Welfare Policy Research Grants from the Ministry of Health, Labour, and Welfare of Japan (Policy Research for Hepatitis Measures [grant number 23HC2002]).

## Ethics Statement

The study was conducted in accordance with the Declaration of Helsinki. The survey was conducted anonymously, and participation was voluntary. Electronic informed consent was obtained from all participants through voluntary completion and submission of the questionnaire. No minors were included in this study. The study protocol was approved by the Institutional Review Board of Saga University Faculty of Medicine.

## Conflicts of Interest

The authors declare no conflicts of interest.

## Supporting information


**Figure S1:** Study design of the prospective survey. Banner advertisement was shown for the people when they searched for key terms (KTG) and for people when they searched any words (CTR). CTR, control; KTG, key term group.
**Table S1:** Click‐through rate of the banner advertisement.
Click‐through rate of the banner advertisement was compared between KTG and CTR. *p = 0.001 and **p < 0.001 by Fisher's exact test. CTR, control; KTG, key term group; y.o., years old.
**Table S2:** Search terms prior to searching for “fatty liver” and “how to treat.” The table shows the timing and search volume of terms searched before users searched for “fatty liver” and “how to treat.”

## Data Availability

The data that support the findings of this study are available on request from the corresponding author. The data are not publicly available due to privacy or ethical restrictions.
