# Supplementary material for: Novel Internet Advertising Approach to Raise Public Awareness About Metabolic Dysfunction‐Associated Steatotic Liver Disease
Source: JGH Open. 2026 Jun 20;10(6):e70436. doi: 10.1002/jgh3.70436 (PMC13282842; doi:10.1002/jgh3.70436)
Supplement: Supplementary file 1 — Figure S1: Study design of the prospective survey. Banner advertisement was shown for the people when they searched for key terms (KTG) and for people when they searched any words (CTR). CTR, control; KTG, key term group. Table S1: Click‐through rate of the banner advertisement. Click‐through rate of the banner advertisement was compared between KTG and CTR. *p = 0.001 and **p < 0.001 by Fisher's exact test. CTR, control; KTG, key term group; y.o., years old. Table S2: Search terms prior to searching for “fatty liver” and “how to treat.” The table shows the timing and search volume of terms searched before users searched for “fatty liver” and “how to treat.” [file JGH3-10-e70436-s001.docx]

**Supplementary File**

Figure S1. Study design of the prospective survey.


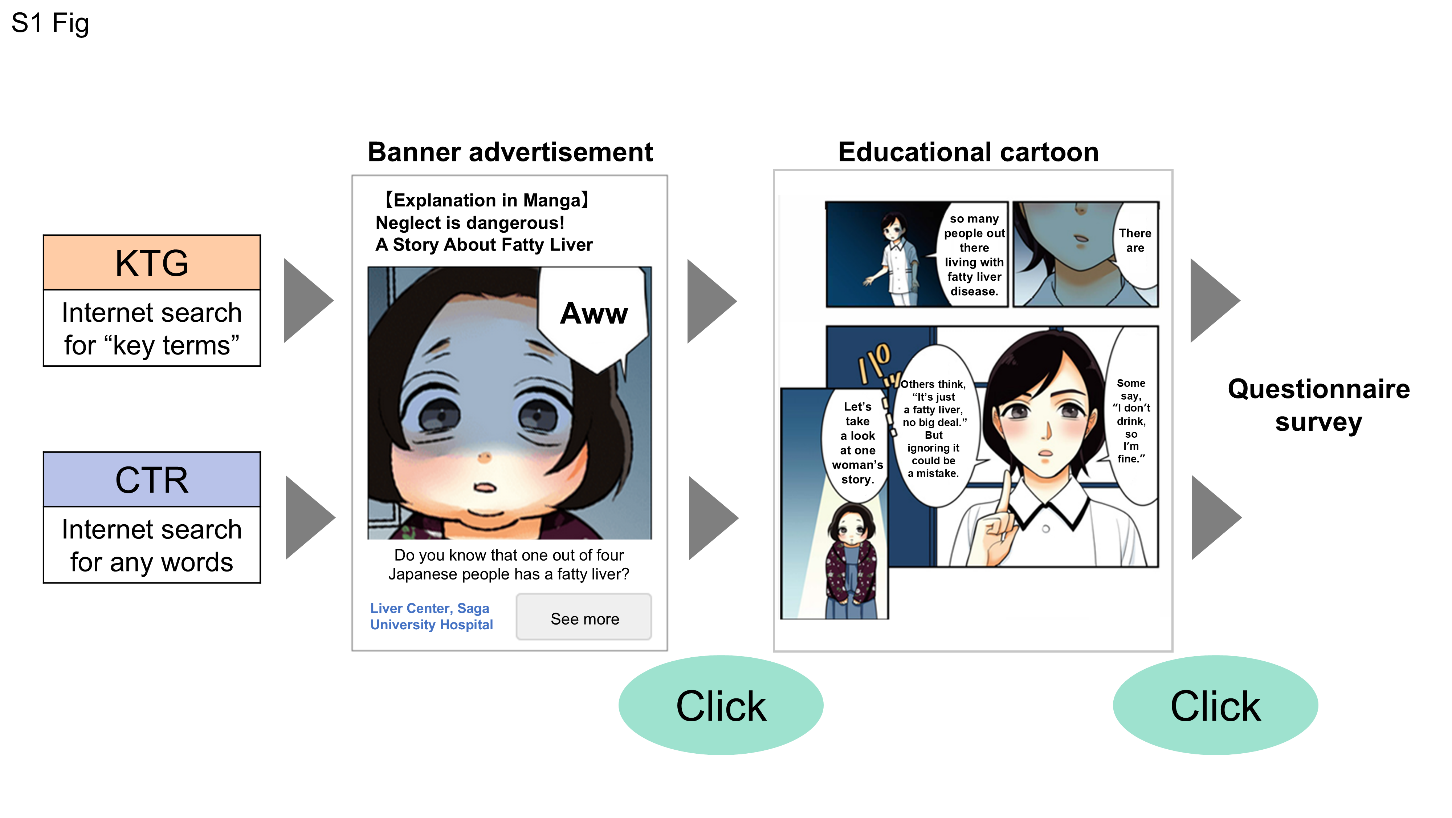


Banner advertisement was shown for the people when they searched for key terms (KTG) and for people when they searched any words (CTR). KTG, key term group; CTR, control.

Table S1. Click-through rate of the banner advertisement

|  | KTG | | | CTR | | |  |
| --- | --- | --- | --- | --- | --- | --- | --- |
| Age | Impression (n) | Click (n) | Click through rate (%) | Impression (n) | Click (n) | Click through rate (%) | p value |
| Overall | 5,864,184 | 5,675 | 0.097 | 49,388,176 | 31,153 | 0.063 | < 0.001 |
| Female Overall | 2,910,164 | 3,427 | 0.118 | 11,481,853 | 10,874 | 0.095 | < 0.001 |
| 40 - 44 y.o | 909,925 | 949 | 0.104 | 4,106,592 | 3,625 | 0.088 | < 0.001 |
| 45 - 49 y.o | 932,762 | 1,101 | 0.118 | 4,142,313 | 3,832 | 0.093 | < 0.001 |
| 50 - 54 y.o | 630,551 | 801 | 0.127 | 1,882,712 | 2,033 | 0.108 | < 0.001 |
| 55 - 59 y.o | 436,926 | 574 | 0.131 | 1,350,236 | 1,384 | 0.103 | < 0.001 |
| Male Overall | 2,954,020 | 2,248 | 0.076 | 37,906,323 | 20,279 | 0.053 | 0.001 |
| 40 - 44 y.o | 837,103 | 619 | 0.074 | 10,167,447 | 4,569 | 0.040 | < 0.001 |
| 45 - 49 y.o | 937,132 | 712 | 0.076 | 10,487,697 | 5,351 | 0.050 | < 0.001 |
| 50 - 54 y.o | 670,365 | 519 | 0.077 | 10,140,140 | 5,695 | 0.060 | < 0.001 |
| 55 - 59 y.o | 509,420 | 398 | 0.078 | 7,121,039 | 4,664 | 0.070 | < 0.001 |

Click-through rate of the banner advertisement was compared between KTG and CTR. *p=0.001 and **p<0.001 by Fisher’s exact test. KTG, key terms group; CTR, control; y.o, years old.

Table S2. Search terms prior to searching for “fatty liver” and “how to treat.

| Search term | Day before standard term search | Search volume |
| --- | --- | --- |
| How to do reverse crunches | -147.809 | 60 |
| Adebose kowa granules 10 | -139.101 | 60 |
| What is peritoneal cancer? | -99.091 | 70 |
| Types of antihypertensive drugs | -97.184 | 80 |
| Foaming urine in diabetes | -96.916 | 60 |
| Symptoms of peripheral artery disease | -83.488 | 110 |
| Cabbage diet methods and effects | -71.528 | 110 |
| List of hypertension medication | -69.11 | 70 |
| Tea to reduce bad cholesterol | -68.544 | 60 |
| Diet methods for people aged 50 and above | -63.457 | 70 |
| Pain under the right scapula | -63.023 | 70 |
| After breast cancer surgery | -59.406 | 170 |
| Bubbles in Urine in Photos with Diabetes | -56.431 | 240 |
| Diseases causing pain in the right back | -55.879 | 100 |
| Effects of gazelle bacteria | -55.422 | 140 |
| Over-the-counter drugs for psoriasis | -53.648 | 100 |
| Health benefits of W's green juice | -47.79 | 70 |
| Maitake mushroom diet method | -46.936 | 170 |
| Foods high in branched-chain fatty acids | -44.037 | 180 |
| Pain in the armpit and abdomen | -43.513 | 60 |
| Large size women's clothing | -43.256 | 80 |
| Side effects of Bofutsusho-san (herbal medicine) | -40.649 | 330 |
| Soup broth diet methods | -40.174 | 110 |
| Vinegar for dieting | -39.536 | 60 |
| Improving kidney function | -39.02 | 150 |
| Shortness of breath when climbing stairs | -38.533 | 60 |
| Acupressure points for frozen shoulder | -38.284 | 70 |
| Shibohiru Gold | -38.203 | 110 |
| Losing weight in your 50s | -37.722 | 60 |
| Symptoms of sudden sweating | -37.62 | 100 |
| Successful diet methods for people in their 50s | -37.305 | 190 |
| Diet clinics | -35.953 | 370 |
| Blog on uterine body cancer | -35.914 | 70 |
| Effect of Tsumura 12 | -35.73 | 60 |
| Ways to make blood vessels flexible | -35.293 | 70 |
| Fat-dissolving cream | -35.181 | 60 |
| Micro diet for breakfast replacement | -35.018 | 90 |
| Table S2 (continued) | | |
| What is Shirota strain? | -34.571 | 80 |
| Dark Urine Color | -33.118 | 290 |
| Inflammation in the throat that won't heal | -32.481 | 60 |
| Beni-no-iro | -31.906 | 110 |
| CA19-9 tumor marker values | -31.543 | 80 |
| Black sesame barley tea lowers blood pressure | -31.084 | 80 |
| Symptoms of gallbladder inflammation | -30.109 | 90 |
| White tongue coating causes | -29.947 | 60 |
| Treatment for chronic gastritis | -29.682 | 150 |
| Causes of throbbing headaches | -29.315 | 70 |
| Pain on the right side of the abdomen, below the ribs | -29.254 | 100 |
| 1-week menu for low-carbohydrate diets | -29.074 | 110 |
| People who are prone to breast cancer recurrence and metastasis | -28.093 | 180 |
| Side Effects of Tamoxifen | -26.522 | 240 |
| What are probing fatty acids? | -26.425 | 80 |
| GLP-1 supplements | -26.39 | 70 |
| Side effects of Xeloda | -26.159 | 80 |
| Diseases that cause weight gain | -25.957 | 190 |
| Ways to improve fluid metabolism | -25.872 | 70 |
| Foamy urine | -24.728 | 130 |
| Gazelle bacterium sp strain yogurt | -24.108 | 70 |
| Surgery for uterine cancer | -24.093 | 60 |
| Diet for high cholesterol | -23.444 | 130 |
| When to Take Naisupport | -23.205 | 260 |
| Pain in the upper part of the stomach | -23.125 | 70 |
| how to make broth for a diet | -22.863 | 620 |
| Dress code for niece's wedding | -22.607 | 60 |
| Benefits of koji water | -22.307 | 110 |
| Tsumura 107 Goshajinkigan | -22.071 | 90 |
| Effects of NaiShi Support | -21.461 | 130 |
| Alkaline phosphatase | -21.252 | 60 |
| Early symptoms of stomach cancer for women | -20.998 | 70 |
| Insurance coverage for cancer patients | -20.27 | 90 |
| Tumor marker values | -20.037 | 150 |
| Momota Buko blog | -19.929 | 150 |
| Koji diet | -19.446 | 60 |
| Foods containing inulin | -19.124 | 70 |
| Effects and benefits of onions | -18.626 | 60 |
| Menopausal headaches | -18.41 | 80 |
| Diseases that cause pain in the right side of the waist | -18.155 | 110 |
| Table S2 (continued) | | |
| Causes of High Diastolic Blood Pressure | -17.52 | 310 |
| Ways to lower lower blood pressure | -17.473 | 150 |
| How to remove water from the knee | -17.447 | 80 |
| Insurance coverage for obesity | -17.376 | 160 |
| Kidney tumors | -17.322 | 80 |
| Ways to lower CA19-9 | -17.129 | 100 |
| High diastolic blood pressure | -16.546 | 70 |
| Can lactic acid bacteria help in losing weight? | -16.155 | 80 |
| Hemoglobin A1C reference values | -16.034 | 90 |
| Treatment for pancreatitis | -15.73 | 200 |
| Diet method for people in their 50s | -15.365 | 70 |
| Rapid weight gain | -14.918 | 130 |
| Symptoms of Diabetes in Women | -14.654 | 260 |
| Feeling of heat in the stomach | -14.558 | 130 |
| Improvement of thick and sticky blood | -14.279 | 60 |
| Discomfort on the Left Flank | -14.143 | 250 |
| Benefits of quitting alcohol | -13.996 | 80 |
| Foods that eliminate cancer cells | -13.81 | 90 |
| Pain from Right Flank to Back | -13.709 | 300 |
| Blog about uterine cancer | -13.449 | 60 |
| Foods that lower uric acid levels | -13.38 | 800 |
| Renal reconstruction surgery | -13.215 | 130 |
| Symptoms of hypothyroidism | -13.152 | 80 |
| How to keep blood thin | -13.11 | 110 |
| Diseases that cause foamy urine | -13.086 | 150 |
| What is Cholangitis? | -13.005 | 220 |
| What is a pancreatic cyst? | -12.73 | 80 |
| Symptoms of Kidney Stones | -12.705 | 260 |
| Pain in the right side of the abdomen | -12.587 | 120 |
| Effectiveness of barley young leaf green juice | -12.483 | 80 |
| Dizziness in diabetes | -12.473 | 60 |
| Foods that lower LDL cholesterol | -12.199 | 210 |
| Menopausal pain in the upper arm | -12.182 | 60 |
| Pain on the right side of the back | -12.114 | 200 |
| Diseases that cause weight gain | -12.026 | 180 |
| Tobacco and blood pressure | -11.392 | 50 |
| Ways to reduce belly fat | -11.315 | 210 |
| Presence of occult blood in urine | -11.305 | 60 |
| Effective ways to drink konbu water | -11.278 | 90 |
| Losing belly fat | -11.189 | 60 |
| Table S2 (continued) | | |
| Naisi support | -11.181 | 1100 |
| Coffee to lower blood sugar | -11.061 | 70 |
| Effective ways to use fitness bikes | -11.035 | 70 |
| What is Ascites? | -11.012 | 270 |
| How to lower cholesterol levels | -10.998 | 1100 |
| Rosuvastatin tablet 2.5 | -10.862 | 450 |
| Urine sugar level 4+ | -10.797 | 70 |
| Support for visceral fat reduction | -10.772 | 160 |
| high triglycerides | -10.494 | 640 |
| Treatment for polycythemia | -10.47 | 50 |
| Side effects of Feburic | -10.37 | 70 |
| High fasting blood sugar levels | -10.302 | 90 |
| Ways to quit drinking alcohol | -10.131 | 130 |
| Benefits of abstaining from alcohol | -10.071 | 210 |
| Primary biliary cholangitis | -9.99 | 130 |
| Types of blood pressure medication | -9.852 | 70 |
| Dull pain in the right abdomen | -9.62 | 90 |
| Reasons for high blood pressure | -9.598 | 180 |
| Health luck in feng shui | -9.304 | 60 |
| the benefits of onion vinegar | -9.172 | 390 |
| Whitish stools | -9.167 | 130 |
| Side effects of Zechia tablets (10mg) | -9.064 | 100 |
| Diseases that cause left waist pain | -8.986 | 110 |
| Hypothyroidism causes weight gain | -8.966 | 80 |
| Dealing with gout attacks | -8.965 | 100 |
| Causes of protein in urine | -8.823 | 170 |
| Abdominal CT scan | -8.783 | 100 |
| Drinks for diabetes | -8.71 | 70 |
| Side effects of CPAP (continuous positive airway pressure) | -8.69 | 100 |
| Gallbladder Surgery | -8.611 | 300 |
| How to lose 20 kilograms | -8.466 | 200 |
| CT scan and meals | -8.409 | 120 |
| Diet and abstaining from alcohol | -8.158 | 80 |
| When the liver becomes unhealthy | -8.081 | 100 |
| Valmodia | -7.829 | 70 |
| Obesity clinic | -7.806 | 770 |
| Blood pressure app | -7.688 | 80 |
| symptoms of kidney cancer | -7.683 | 450 |
| Pain on the right side of the navel | -7.625 | 140 |
| Pain in the lower left part of the back | -7.554 | 80 |
| Table S2 (continued) | | |
| How to spend time when blood pressure is high | -7.37 | 150 |
| Joint pain during menopause | -7.206 | 130 |
| Stopping antihypertensive medication | -7.152 | 100 |
| Tea that is effective for diabetes | -7.141 | 80 |
| Anti-cancer diet | -7.027 | 70 |
| Dull pain in the abdomen | -6.941 | 130 |
| How long it takes for the effects of walking to show | -6.872 | 60 |
| Reviews of Kaitousha tea | -6.751 | 820 |
| Tomato juice for diabetes | -6.604 | 160 |
| Pain on the right side of the back | -6.523 | 160 |
| Early symptoms of diabetes | -6.447 | 120 |
| Diseases where pain moves around | -6.341 | 90 |
| Renowned gastroenterologists | -6.319 | 120 |
| Feeling unwell due to being overweight | -6.308 | 120 |
| End-stage liver cirrhosis | -6.149 | 110 |
| Recipes for diabetes-friendly meals | -6.127 | 60 |
| Obesity clinic in Tokyo | -6.077 | 100 |
| Terminalia First reviews and side effects | -5.979 | 70 |
| Rice and carbohydrates | -5.961 | 60 |
| Medications for lowering cholesterol | -5.944 | 80 |
| How to lower vascular age | -5.855 | 860 |
| Cold weather raises blood pressure | -5.82 | 110 |
| Tsumura 25 effects | -5.767 | 110 |
| Organs under the ribcage | -5.647 | 70 |
| Life expectancy for liver cancer | -5.529 | 80 |
| What is cholecystitis? | -5.514 | 90 |
| Effects of clams | -5.469 | 210 |
| Pain on the right side of the abdomen | -5.455 | 150 |
| Blood in stool | -5.411 | 60 |
| Ways to quit drinking alcohol | -5.361 | 150 |
| Symptoms of high liver values | -5.345 | 180 |
| Foods and drinks that dissolve blood clots | -5.341 | 120 |
| Ways to reduce tongue swelling | -5.333 | 70 |
| Symptoms of hepatitis C | -5.099 | 90 |
| Symptoms of Gastroesophageal Reflux Disease (GERD) | -5.061 | 290 |
| pain in the upper right back | -5.011 | 410 |
| Bottled Duzhong tea | -4.598 | 80 |
| Kidney supplements | -4.546 | 80 |
| Effectiveness of Shiboheer | -4.502 | 90 |
| Wild Mango Force reviews | -4.406 | 70 |
| Table S2 (continued) | | |
| Pain on the left side of the epigastric region | -4.368 | 180 |
| Pulmonary nodules | -4.306 | 70 |
| Losing weight from 80 kg | -4.23 | 80 |
| what is gallstones | -4.117 | 490 |
| Acupressure points to lower blood pressure | -4.079 | 80 |
| how to quickly lose visceral fat | -4.055 | 410 |
| Supplements that lower blood sugar levels | -4.019 | 100 |
| Pain from the throat to the chest | -3.994 | 60 |
| Postprandial blood sugar | -3.964 | 130 |
| Symptoms of Chronic Pancreatitis | -3.891 | 260 |
| Scars from open surgery | -3.522 | 70 |
| Kidney stones | -3.47 | 150 |
| Symptoms of Ascites | -3.465 | 280 |
| Diet methods for people in their 50s | -3.413 | 900 |
| Kenjin's green tea | -3.379 | 60 |
| Treatment of liver cancer | -3.378 | 120 |
| Causes of high morning blood pressure | -3.358 | 90 |
| Contrast-enhanced CT scan | -3.284 | 80 |
| Causes of High Liver Function Test Results | -3.255 | 260 |
| Protruding upper abdomen | -3.199 | 180 |
| Effectiveness of abstinence from alcohol | -3.085 | 1000 |
| Complete cure for diabetes | -3.083 | 170 |
| gallstones | -3.016 | 320 |
| Rejuvenating lung age | -3 | 70 |
| Kidney stone pain | -2.972 | 70 |
| Effectiveness of Tsumura 62 | -2.886 | 80 |
| red palms | -2.859 | 610 |
| High lipase levels | -2.789 | 70 |
| Can stopping alcohol consumption help with weight loss? | -2.743 | 100 |
| What Can be Found in Abdominal Ultrasound Tests | -2.697 | 280 |
| what is ALP | -2.595 | 420 |
| causes of high diastolic blood pressure | -2.564 | 620 |
| Urashirogashi tea | -2.504 | 150 |
| Effectiveness and reviews of Naishi support | -2.459 | 840 |
| liver symptoms | -2.447 | 360 |
| Pancreatic testing | -2.404 | 140 |
| Metabolic syndrome | -2.315 | 80 |
| High fasting blood sugar levels cause | -2.264 | 70 |
| Tamoxifen causes weight gain | -2.218 | 100 |
| Foods that strengthen blood vessels | -2.209 | 680 |
| Table S2 (continued) | | |
| Symptoms of Bile Duct Cancer | -2.206 | 300 |
| Diseases causing weight gain | -2.205 | 100 |
| What are liver function tests? | -2.176 | 90 |
| What is dyslipidemia | -2.15 | 730 |
| Pain on the lower right side of the ribcage | -2.144 | 210 |
| How to reduce visceral fat in men | -2.11 | 80 |
| Holly P of the stomach | -2.053 | 70 |
| Liver Function Tests | -2.053 | 220 |
| diets to lower triglycerides | -2.021 | 620 |
| Cancer detected in blood tests | -1.913 | 130 |
| Diseases that Cause Abdominal Distention | -1.847 | 240 |
| Pain in the right hypochondrium | -1.823 | 70 |
| What are kidney stones? | -1.752 | 110 |
| Akapori | -1.563 | 50 |
| Gallbladder pain | -1.546 | 190 |
| Complete cure for hepatitis C | -1.544 | 60 |
| Withdrawal symptoms from alcohol | -1.538 | 100 |
| health check-ups and re-examinations | -1.532 | 330 |
| Pancreatic cysts | -1.49 | 190 |
| Discomfort on the Right Flank | -1.49 | 270 |
| Information obtained from ultrasound examination | -1.41 | 160 |
| Foods that are good for the liver | -1.356 | 140 |
| Treatment for hypercholesterolemia | -1.355 | 60 |
| Immediate ways to lower blood pressure | -1.323 | 140 |
| Causes of high MCV and MCH levels | -1.266 | 70 |
| Urticaria in the liver | -1.242 | 100 |
| Dull pain in the left side of the abdomen | -1.229 | 70 |
| improving liver function | -1.227 | 660 |
| Symptoms of liver damage | -1.172 | 130 |
| Blood test for pancreatic function | -1.151 | 60 |
| Liver function tests | -1.13 | 120 |
| Body fat percentage for women in their 50s | -1.066 | 90 |
| Diet method for intestinal flora | -1.033 | 60 |
| liver function values | -1.026 | 680 |
| What Type of Specialist is Needed for Liver Problems | -1.022 | 240 |
| Noom pricing | -1.014 | 140 |
| Symptoms of gallstones | -0.993 | 3100 |
| Lactobacillus Yogurt | -0.985 | 250 |
| Diseases diagnosed by gastric camera | -0.984 | 70 |
| Side effects of drinking too much lemon water | -0.98 | 70 |
| Table S2 (continued) | | |
| Gamma marker values | -0.978 | 80 |
| Abdominal ultrasound examination | -0.969 | 100 |
| Pain in the right upper abdomen | -0.955 | 110 |
| Early Symptoms of Gallbladder Cancer | -0.932 | 220 |
| Symptoms of pancreatic cancer in the early stages | -0.916 | 80 |
| Blood glucose test | -0.907 | 70 |
| Causes of sudden high blood pressure | -0.9 | 120 |
| Pain Under the Right Rib Cage | -0.893 | 250 |
| Eosinophils | -0.829 | 130 |
| Dietary Treatment for Pancreatitis | -0.827 | 290 |
| pancreatic pain | -0.813 | 390 |
| Lactoferrin effects | -0.808 | 1000 |
| Prediabetes | -0.801 | 150 |
| Foamy Urine | -0.795 | 230 |
| Difference between gastroenterology and digestive medicine | -0.778 | 70 |
| Benefits of quitting alcohol | -0.776 | 80 |
| Causes of high liver function values | -0.752 | 5600 |
| Pain when pressing the right side of the abdomen | -0.742 | 70 |
| Pancreatic cancer testing methods | -0.737 | 140 |
| Medicines for the liver | -0.734 | 110 |
| Hata Internal Medicine Clinic | -0.725 | 50 |
| Causes of high triglycerides | -0.72 | 850 |
| Liver disorders | -0.682 | 150 |
| Blood sugar levels in diabetes | -0.651 | 60 |
| Effect of blue fish | -0.64 | 70 |
| Menopause herbal medicine | -0.634 | 70 |
| Hepatitis virus | -0.625 | 140 |
| Liver function | -0.603 | 760 |
| Levawulso | -0.602 | 70 |
| Gamma-glutamyl Transferase (GGT) | -0.592 | 240 |
| abdominal ultrasound examination | -0.574 | 400 |
| after eradication of Helicobacter pylori | -0.547 | 380 |
| Gallstones | -0.544 | 70 |
| Can diabetes be cured? | -0.515 | 100 |
| Cost of abdominal ultrasound | -0.5 | 150 |
| Harvard-style vegetable soup | -0.496 | 80 |
| Cancer detected in pet tests | -0.488 | 150 |
| Which department to visit for gallstones | -0.423 | 180 |
| Liver Abnormalities on Health Checkup | -0.418 | 230 |
| GABA tea | -0.414 | 100 |
| Table S2 (continued) | | |
| Gallstone pain | -0.414 | 200 |
| What is liver cirrhosis | -0.404 | 720 |
| Colon Holly up | -0.398 | 70 |
| High liver values in blood tests | -0.395 | 180 |
| Drinking alcohol and health | -0.378 | 120 |
| High total protein levels | -0.361 | 70 |
| Foods that are bad for the liver | -0.344 | 210 |
| Liver function in health checkups | -0.342 | 190 |
| Abdominal ultrasound and diet | -0.341 | 160 |
| Symptoms of acute hepatitis | -0.332 | 100 |
| Common bile duct dilation | -0.314 | 70 |
| Abdominal ultrasound | -0.311 | 690 |
| What can be learned from abdominal ultrasonography | -0.307 | 190 |
| Liver dysfunction | -0.304 | 2000 |
| Gamma-GTP | -0.295 | 1600 |
| ALT Liver Function | -0.291 | 320 |
| Effectiveness of kudzu tea | -0.285 | 160 |
| Pain in the upper left abdomen | -0.279 | 200 |
| Medications for the liver | -0.242 | 200 |
| Foods that lower HbA1c levels | -0.235 | 80 |
| Effectiveness of walking 30 minutes every day | -0.228 | 70 |
| how to quit drinking | -0.221 | 430 |
| Viral hepatitis | -0.198 | 150 |
| Early-stage liver cancer | -0.189 | 140 |
| Symptoms of liver cancer | -0.183 | 150 |
| Detailed health examination | -0.182 | 70 |
| High liver values | -0.173 | 9100 |
| High gamma-GTP levels | -0.172 | 6300 |
| Shadows on the liver | -0.167 | 110 |
| Postprandial blood glucose level | -0.162 | 460 |
| Diabetes and carbohydrate restriction | -0.16 | 70 |
| What is erosive gastritis? | -0.156 | 100 |
| Liver blood test | -0.152 | 100 |
| Symptoms of liver cancer | -0.143 | 4100 |
| Diet for cholecystitis | -0.141 | 140 |
| Drinks that are good for the kidneys | -0.129 | 60 |
| Causes of High ALT Levels | -0.126 | 260 |
| How to lower blood sugar levels | -0.125 | 1900 |
| Foods high in taurine | -0.12 | 110 |
| What is acute hepatitis? | -0.116 | 210 |
| Table S2 (continued) | | |
| Causes of high liver values | -0.114 | 170 |
| GOT in blood tests | -0.111 | 100 |
| causes of high R-GTP levels | -0.109 | 430 |
| Dangers of Ninjin-tan’nichi | -0.106 | 320 |
| Blood sugar supplements | -0.104 | 90 |
| TG blood test | -0.094 | 510 |
| Pain on the right side of the ribcage | -0.093 | 80 |
| Ultrasound examination | -0.093 | 690 |
| High levels of RGTP | -0.09 | 160 |
| Need for detailed health examination | -0.089 | 90 |
| AFP tumor marker | -0.088 | 320 |
| Survival rate for gallbladder cancer | -0.071 | 120 |
| GPT reference value | -0.067 | 160 |
| Ways to keep kidneys healthy | -0.066 | 70 |
| GTP reference values | -0.063 | 90 |
| Pancreatic diseases | -0.059 | 1400 |
| Meaning of heart shadow expansion | -0.049 | 70 |
| Pain in the right back | -0.047 | 850 |
| Liver Tumors | -0.045 | 270 |
| R-GT | -0.041 | 80 |
| R-GTP | -0.041 | 80 |
| Medicines that dissolve fat | -0.04 | 100 |
| Bile duct | -0.035 | 80 |
| Symptoms of Liver Disease | -0.033 | 270 |
| Hepatitis virus test | -0.033 | 490 |
| what can be diagnosed with abdominal echo | -0.031 | 390 |
| Chronic gastritis without Helicobacter pylori | -0.03 | 80 |
| Liver diseases | -0.028 | 2200 |
| High IgG levels | -0.027 | 80 |
| what is hepatitis | -0.027 | 680 |
| Location of the liver | -0.025 | 200 |
| Treatment for Gallstones | -0.024 | 270 |
| Blood tests for liver function | -0.023 | 110 |
| What Can be Found in Abdominal CT Scans | -0.022 | 220 |
| ALT | -0.022 | 3400 |
| symptoms of liver dysfunction | -0.021 | 530 |
| GPT liver function test | -0.02 | 120 |
| Liver tumor | -0.02 | 140 |
| Blog about lung cancer | -0.019 | 120 |
| Obesity-related diseases | -0.019 | 130 |
| Table S2 (continued) | | |
| What is R-GTP? | -0.018 | 100 |
| High AST/ALT Levels | -0.018 | 310 |
| GOT | -0.018 | 970 |
| Causes of high GPT levels | -0.018 | 2300 |
| High ALT Levels | -0.017 | 230 |
| What is Cholinesterase? | -0.017 | 270 |
| end-stage liver cancer | -0.015 | 520 |
| Liver disease | -0.014 | 140 |
| Liver Hemangioma | -0.014 | 240 |
| causes of high GOT and GPT levels | -0.014 | 400 |
| Symptoms of hepatitis C | -0.012 | 110 |
| High levels of gamma-GTP | -0.012 | 150 |
| what is GPT | -0.012 | 410 |
| Liver cancer | -0.012 | 750 |
| location of the organs | -0.011 | 350 |
| high GTP levels | -0.011 | 540 |
| Liver cancer | -0.01 | 130 |
| Reference Values for AST | -0.01 | 300 |
| Being told that blood is thick | -0.009 | 80 |
| What are gallstones? | -0.008 | 120 |
| Medications for gallstones | -0.008 | 120 |
| High levels of ALT in blood tests | -0.008 | 140 |
| How to take liver rest days | -0.008 | 170 |
| Supplements to lower cholesterol | -0.008 | 180 |
| Effects of Quitting Alcohol | -0.008 | 290 |
| decreased liver function | -0.008 | 380 |
| High AST levels | -0.008 | 1900 |
| Foods that are good for the liver | -0.008 | 6600 |
| Location of the liver | -0.007 | 90 |
| Can Diabetes be Cured? | -0.007 | 300 |
| Liver cancer surgery | -0.006 | 80 |
| Liver-friendly foods | -0.006 | 120 |
| High liver enzymes | -0.006 | 140 |
| AST/ALT Levels | -0.006 | 240 |
| Liver Cancer Survival Rate | -0.006 | 320 |
| How to reduce liver fat | -0.006 | 990 |
| Symptoms of atrophic gastritis | -0.005 | 70 |
| Foods rich in ornithine | -0.005 | 80 |
| What is TG in blood tests? | -0.005 | 90 |
| Symptoms of renal cyst | -0.005 | 140 |
| Table S2 (continued) | | |
| How to improve liver health | -0.005 | 160 |
| Pain in the Upper Right Abdomen | -0.005 | 240 |
| causes of high gamma GTP levels | -0.005 | 360 |
| chronic hepatitis | -0.005 | 360 |
| Symptoms of fatty liver | -0.005 | 480 |
| AST blood test | -0.004 | 70 |
| What is a liver hematoma? | -0.004 | 90 |
| Suspected liver hemangioma | -0.004 | 100 |
| Gamma-glutamyl transpeptidase | -0.004 | 130 |
| Symptoms of liver failure | -0.004 | 150 |
| Symptoms of decreased liver function | -0.004 | 150 |
| What is hepatic dysfunction? | -0.004 | 200 |
| gamma-GT | -0.004 | 330 |
| CHE (cholinesterase) | -0.004 | 350 |
| Liver pain | -0.004 | 960 |
| AST | -0.004 | 1000 |
| How to reduce visceral fat | -0.004 | 1400 |
| How many days are required for a break in publication? | -0.003 | 70 |
| Itchy body, liver problem | -0.003 | 70 |
| Causes of liver cysts | -0.003 | 110 |
| Life expectancy with liver cancer | -0.003 | 120 |
| High levels of cholinesterase | -0.003 | 120 |
| Causes of gallbladder polyps | -0.003 | 140 |
| Symptoms of Kidney Stones | -0.003 | 230 |
| What is Hepatic Steatosis? | -0.003 | 250 |
| Symptoms of Liver Damage | -0.003 | 290 |
| gallbladder adenomyoma | -0.003 | 340 |
| lowering gamma GTP | -0.003 | 370 |
| how to lower liver function values | -0.003 | 430 |
| Cholinesterase | -0.003 | 720 |
| Liver | -0.003 | 2600 |
| High ALT levels | -0.003 | 3100 |
| Curable aspects of diabetes | -0.002 | 70 |
| What is gastric erosion? | -0.002 | 70 |
| Abdominal aortic calcification | -0.002 | 70 |
| Liver function blood test | -0.002 | 90 |
| Liver cyst | -0.002 | 100 |
| What is a liver cyst | -0.002 | 130 |
| What can be determined through CT scans | -0.002 | 150 |
| How to reduce visceral fat | -0.002 | 150 |
| Table S2 (continued) | | |
| What is a hepatic cyst? | -0.002 | 180 |
| Medications that are effective for the liver | -0.002 | 190 |
| Liver recovery | -0.002 | 220 |
| Ascites in Liver Cirrhosis | -0.002 | 250 |
| hepatitis treatment | -0.002 | 330 |
| liver function values | -0.002 | 420 |
| gpt | -0.002 | 500 |
| liver test results | -0.002 | 600 |
| what hepatic hemangioma is | -0.002 | 620 |
| Hepatitis | -0.002 | 1100 |
| Gallbladder polyps | -0.002 | 2800 |
| Liver cirrhosis | -0.002 | 3300 |
| What is fatty liver? | -0.002 | 4000 |
| Effect of a walking machine | -0.001 | 70 |
| Causes of hepatitis | -0.001 | 70 |
| Duodenal polyps | -0.001 | 90 |
| Treatment for kidney stones | -0.001 | 90 |
| Dull pain in the right side of the abdomen | -0.001 | 90 |
| Liver diseases | -0.001 | 100 |
| Ways to improve diabetes | -0.001 | 110 |
| What is HBs antigen | -0.001 | 150 |
| What is splenomegaly | -0.001 | 150 |
| Causes of liver cirrhosis | -0.001 | 170 |
| Itching of the liver | -0.001 | 170 |
| Liver values | -0.001 | 190 |
| What is gastric low-grade dysplasia? | -0.001 | 210 |
| Liver Cysts | -0.001 | 300 |
| gallstones | -0.001 | 510 |
| Gamma-GTP | -0.001 | 770 |
| Restoring liver function | -0.001 | 1300 |
| Symptoms of hepatitis | -0.001 | 1600 |
| High ALP levels | -0.001 | 1800 |
| Converting fat into muscle | 0 | 70 |
| What is aortic calcification? | 0 | 90 |
| Bacteria that digest fat | 0 | 90 |
| Foods that burn fat | 0 | 110 |
| Liver tumors | 0 | 140 |
| Natural cure for lipoma | 0 | 210 |
| Images of Lipomas | 0 | 240 |
| Renal Calcification | 0 | 240 |
| Table S2 (continued) | | |
| Diseases of the Spleen | 0 | 250 |
| Pain in the Liver Area | 0 | 250 |
| Alcoholic Liver Disease | 0 | 280 |
| fat | 0 | 350 |
| how to remove fat deposits | 0 | 360 |
| lipoma surgery | 0 | 400 |
| acute hepatitis | 0 | 430 |
| what is a lipoma | 0 | 450 |
| What is fatty stool | 0 | 460 |
| Hepatic lipoma | 0 | 2600 |
